# Grain width 2 (GW2) and its interacting proteins regulate seed development in rice (*Oryza sativa* L.)

**DOI:** 10.1186/s40529-018-0240-z

**Published:** 2018-10-26

**Authors:** Kyu Ho Lee, Sang Woo Park, Yeon Jeong Kim, Yeon Jong Koo, Jong Tae Song, Hak Soo Seo

**Affiliations:** 1 Department of Plant Science, College of Agriculture and Life Sciences, Research Institute of Agriculture and Life Sciences, Seoul National University, Gwanakro 200, Gwanak-gu, Seoul, 151-921 South Korea; 2 Department of Biological Chemistry, Chonnam National University, Gwangju, 61186 South Korea; 3 School of Applied Biosciences, Kyungpook National University, Daegu, 41566 South Korea

**Keywords:** Aleurone layer, CHT14, E3 ubiquitin ligase, GW2, PDIL1-1, PGK, Rice, Seed size

## Abstract

**Background:**

Seed size has been extensively studied in crop plants, as it determines crop yield. However, the mechanism of seed development remains elusive. In this study, we explored the mechanism of seed development in rice (*Oryza sativa* L.), and identified proteins affecting seed size.

**Results:**

Proteomic analysis showed that glyceraldehyde 3-phosphate dehydrogenase, chitinase 14 (CHT14), and phosphoglycerate kinase (PGK) accumulated to high levels in the seeds of the natural *japonica* rice mutant Oochikara, which carries a loss-of-function mutation in the *grain width 2* (*GW2*) gene; *GW2* encodes a RING-type E3 ubiquitin ligase. In vitro pull-down and ubiquitination assays showed that CHT14 and PGK directly interacted with GW2 but were not ubiquitinated by GW2. Immunoblot analysis revealed that protein disulfide isomerase-like 1-1 accumulated to high levels in young developing seeds of the *gw2* mutant compared with the wild type. Histochemical *β*-*glucuronidase* staining showed strong expression of *GW2* in leaf and root tissues but weak expression in leaf sheaths and internodes. In addition, transformation of the *green fluorescent protein* (*GFP*) gene under the control of the *GW2* promoter in rice revealed *GFP* expression in the aleurone layer of seeds.

**Conclusions:**

Collectively, these results suggest that GW2 regulates seed size through direct interactions with proteins involved in carbohydrate metabolism by modulating their activity or stability and controlling disulfide bond formation in various proteins during seed development. Additionally, GW2 participates in vegetative as well as reproductive growth, and protects the seed from pathogen attack.

## Background

Post-translational modification of proteins involves the covalent addition of functional molecules of proteins, cleavage of protein subunits, or complete degradation of proteins. Ubiquitination is a type of post-translational modification in which the ubiquitin protein is covalently conjugated to the target protein (Ciechanover 1994). Ubiquitination involves three sequential reactions, each catalyzed by three unique enzymes: an ubiquitin-activating enzyme E1, an ubiquitin-conjugating enzyme E2, and a specific ligase E3 (Vierstra 2009; Bie and Ciechanover 2011; Kelley 2018). Ubiquitination is involved in the regulation of protein localization, stability, and activity (Kelley 2018). Target proteins undergo either monoubiquitination or polyubiquitination. The 26S proteasome complex degrades polyubiquitinated proteins but not monoubiquitinated proteins. Monoubiquitination affects the localization and function of the modified protein (Bie and Ciechanover 2011). Ubiquitination is emerging as a key process in the regulation of seed size in crop plants (Song et al. 2007; Choi et al. 2018).

Seed size is a major factor affecting grain yield in rice (*Oryza sativa* L.) (Fan et al. 2006; Bai et al. 2010; Song et al. 2007) and is controlled by the hull. Rice grain width 2 (GW2) is an E3 ubiquitin ligase, which contains a RING finger motif and shows both auto-ubiquitination and substrate ubiquitination activities (Song et al. 2007; Choi et al. 2018). GW2 homologs have been identified in wheat (*Triticum aestivum*; TaGW2) and maize (Zea mays; ZmGW2); these are involved in the regulation of kernel size, weight, width, and maturity (Su et al. 2011; Li et al. 2010). Although progress has been made towards the cloning and characterization of seed size-related quantitative trait loci and genes, mechanisms determining seed size remain unclear. Recently, it was reported that GW2 directly interacts with and ubiquitinates EXPANSIN-LIKE 1 (EXPLA1), thus inactivating it.

Here, we showed that GW2 strongly interacted with chitinase 14 (CHT14) and phosphoglycerate kinase (PGK) but did not ubiquitinate these proteins, suggesting that GW2 regulates seed size control via its interacting partners, CHT14 and PGK. Additionally, the level of protein disulfide isomerase-like 1-1 (PDIL1-1) was higher in young developing seeds of the *gw2* mutant than in the wild-type. Moreover, GW2 was specifically expressed in the aleurone layer. These data suggest that GW2 controls the development of the aleurone layer and the activity or stability of various proteins by regulating disulfide bond formation during seed development.

## Results

### Proteomic analysis of gw2 seeds

**Fig. 1:**
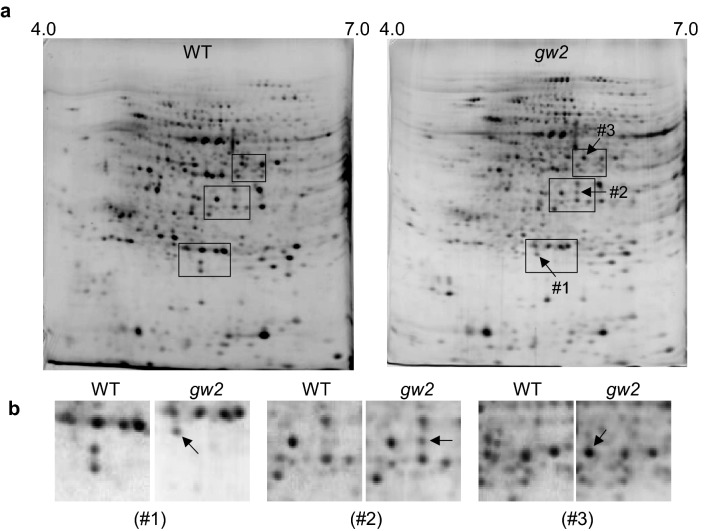
Proteomic analysis of seed proteins in the *grain width 2* (*gw2*) mutant of rice, Oochikara. Total proteins were extracted from whole seeds and subjected to isoelectric focusing (IEF) in the first dimension, using immobilized pH gradient dry strips (pH 4–7 nonlinear, 24 cm in length), and SDS-PAGE in the second dimension, using 10–16% gradient polyacrylamide gels. Gels were stained with silver nitrate. Three independent biological replicates were performed. **a** 2D gels showing protein spots in wild-type and *gw2* mutant rice. Arrowheads indicate highly accumulated proteins in the *gw2* mutant compared with the wild type. Boxed regions shown in **a** are magnified in **b**. Spot #1, glyceraldehyde 3-phosphate dehydrogenase (GAPDH); spot #2, chitinase 14 (CHT14); spot #3, phosphoglycerate kinase (PGK)

Because GW2 protein contains a RING finger domain and has auto-ubiquitination activity, we hypothesized that it functions as an E3 ubiquitin ligase of target proteins involved in GW2-mediated seed development. We used a proteomics approach to identify GW2-interacting proteins. Proteomics is a useful method to identify proteins that accumulate in an organ- or development-specific manner (Cho et al. 2006). Soluble proteins were extracted from the seeds of the wild-type rice cultivar Norin 22 and those of the *japonica* rice *gw2* mutant, Oochikara. These proteins were separated in the first dimension via isoelectric focusing (IEF) on a pH gradient ranging from 4 to 7 and in the second dimension by sodium dodecyl sulfate–polyacrylamide gel electrophoresis (SDS-PAGE) using 10% polyacrylamide gels (Fig. 1a). Protein spots in parallel gels were cross-matched, demonstrating reproducibility. After electrophoresis, gels were analyzed using PDQuest software, and two-dimensional (2-D) gels were systematically compared. Three protein spots showing significant accumulation in *gw2* seeds were selected (Fig. 1a, b). Proteins in these spots were identified via Ettan matrix-assisted laser desorption/ionization time-of-flight mass spectrometry (MALDI-TOF MS), followed by a homology search. The three proteins were identified as glyceraldehyde 3-phosphate dehydrogenase (GAPDH), CHT14, and PGK.

### GW2 interacts with but does not ubiquitinate PGK and CHT14

**Fig. 2:**
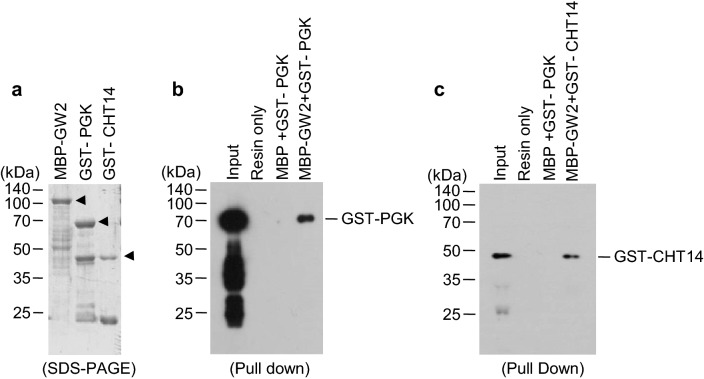
GW2 interacts with PGK and CHT14. **a** Recombinant fusion proteins MBP-GW2, GST-PGK, and GST-CHT14 were overexpressed in *E. coli* and purified using amylose or glutathione resins. Arrowheads indicate MBP-GW2, GST-PGK, and GST-CHT14. **b**, **c** In vitro pull-down assay showing the proteins GST-PGK (**b**) and GST-CHT14 (**c**) pulled down with MBP-GW2, separated on 10% SDS–polyacrylamide gels, and detected by western blotting using anti-GST antibody. *GST* glutathione *S*-transferase, *MBP* maltose-binding protein, *GW2* grain width 2, *PGK* phosphoglycerate kinase, *CHT14* chitinase 14

To characterize GAPDH, PGK, and CHT14, we isolated the corresponding cDNAs using reverse transcriptase PCR (RT-PCR). Gene-specific primers were designed on the basis of sequences present in the nucleotide database of the National Center for Biotechnology Information (NCBI). Results of proteomic analysis showed the accumulation of these proteins in *gw2* seeds, implying that GW2 directly interacts with and possibly ubiquitinates these proteins, thus rendering them liable to degradation by the 26S proteasome complex. Next, we examined the possible interaction of GW2 with each of the three proteins using in vitro pull-down assay. Three recombinant plasmids encoding the maltose-binding protein (MBP) fused with GW2 (MBP-GW2) and glutathione *S*-transferase (GST) fused with CHT14 (GST-CHT14) and PGK (GST-PGK) were constructed and expressed in *Escherichia coli* BL21 (DE3) cells. Recombinant proteins were then affinity purified (Fig. 2a), and GST-CHT14 and GST-PGK were pulled down with either MBP or MBP-GW2. In vitro pull-down assay using MBP-GW2 confirmed that GW2 interacted with CHT14 and PGK (Fig. 2b). However, no interaction was detected between GW2 and GAPDH.

**Fig. 3:**
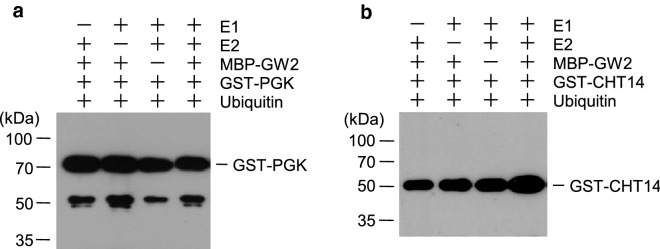
GW2 does not ubiquitinate PGK and CHT14 in vitro. MBP-GW2, GST-PGK, and GST-CHT14 were overexpressed in *E. coli* and purified with amylose or glutathione affinity columns. Ubiquitination of GST-PGK (**a**) or GST-CHT14 (**b**) was examined in the presence or absence of rabbit E1, UbcH5b (E2), MBP-GW2 (E3), and His_6_-ubiquitin. PGK and CHT14 proteins were detected by western blotting using anti-GST antibody

Direct interaction of GW2 with CHT14 and PGK raised the possibility that GW2 may ubiquitinate CHT14 and PGK. However, in vitro ubiquitination assays showed that both CHT14 and PGK were not ubiquitinated (Fig. 3a, b), indicating that GW2 does not ubiquitinate CHT14 and PGK.

### PDIL1-1 accumulates during early seed development in gw2 mutants

**Fig. 4:**
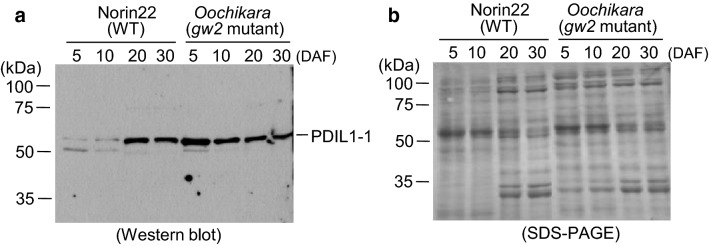
Accumulation of PDIL1-1 protein in *gw2* mutant (Oochikara) and wild-type (Norin 22) seeds during development. Total proteins were extracted from Norin 22 and Oochikara seeds at the indicated time points. After SDS-PAGE, proteins were transferred onto a nitrocellulose membrane. **a** Immunodetection of PDIL1-1 protein using anti-PDIL1-1 antibody. **b** SDS–polyacrylamide gel stained with Coomassie brilliant blue after blotting. PDIL1-1, protein disulfide isomerase-like 1-1

PDIL1-1 regulates the amounts of various seed proteins, which in turn influence the development of endosperm and the aleurone layer (Kim et al. 2012). In addition, seeds of *PDIL1*-*1Δ* exhibit a floury phenotype due to the production of irregular starch granules and protein bodies (Kim et al. 2012). Seeds of the *gw2* mutant also display a floury phenotype (Choi et al. 2018). Therefore, we examined the level of PDIL1-1 during seed development in the *gw2* mutant. Results showed that PDIL1-1 accumulated to high levels in *gw2* seeds at 5 and 10 days after flowering (DAF), whereas its level remained very low in wild-type seeds (Fig. 4a, b), suggesting that GW2 affects the level of PDIL1-1 protein.

### GW2 is highly expressed in panicles and anthers

**Fig. 5:**
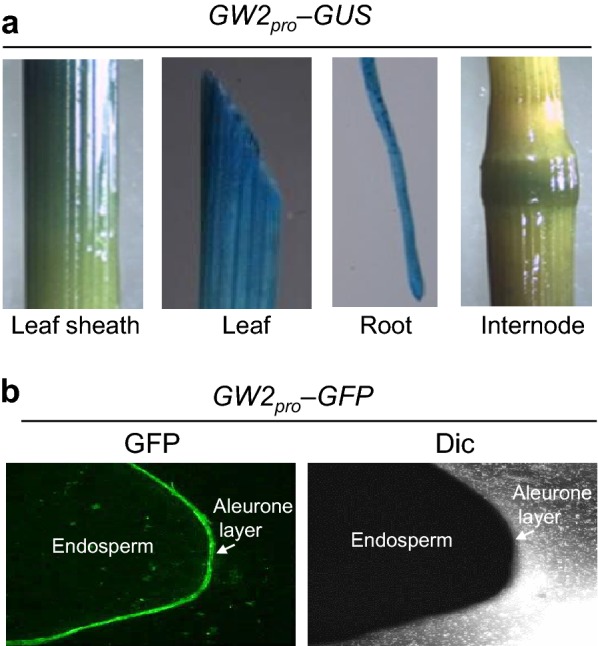
Expression of *GUS* and *GFP* reporter genes under the control of the *GW2* promoter. **a** Examination of *GUS* expression in *GW2*_*pro*_-*GUS* transgenic rice. **b** Detection of GFP fluorescence in *GW2*_*pro*_-*GFP* transgenic rice. GFP imaging was performed using a LAS 4000 imager. *GW2*_*pro*_ *GW2* promoter, *GUS* β-glucuronidase, *GFP* green fluorescent protein

Previous studies showed that *GW2* is expressed in anthers, stamens, pistil, panicles, hull, and endosperm (Song et al. 2007; Choi et al. 2018). To further examine the expression of *GW2*, we generated transgenic rice lines overexpressing *β*-*glucuronidase* (*GUS*) and *green fluorescent protein* (*GFP*) reporter genes under the control of the *GW2* promoter. Histochemical staining of *GW2*_*pro*_-*GUS* plants showed that *GUS* was strongly expressed in leaves and roots but weakly in leaf sheaths and internodes (Fig. 5a). Additionally, fluorescence detection in *GW2*_*pro*_-*GFP* plants revealed a strong GFP signal in the seed aleurone layer (Fig. 5b).

## Discussion

In this study, we show that GW2-interacting proteins, CHT14 and PGK, regulate GW2-mediated seed development. Additionally, GW2 participates in vegetative as well as reproductive growth. Previously, auto-ubiquitination and substrate ubiquitination activities of GW2 have been reported in vitro (Song et al. 2007; Choi et al. 2018), suggesting that the E3 ligase activity of GW2 is required for the regulation of cell size in the spikelet hull. To further study the role of GW2 during seed development, we isolated GW2-interacting proteins whose level and stability were regulated by GW2 activity. Proteomic analysis of *gw2* mutant and wild-type seeds revealed that the levels of several proteins were higher in *gw2* mutant seeds than in wild-type seeds (Fig. 1a, b). Determination of amino acid sequences revealed that GAPDH, CHT14, and PGK proteins accumulated to high levels in *gw2* mutant seeds, suggesting that these proteins are destabilized by the E3 ubiquitin ligase activity of GW2. In vitro pull-down assay showed interaction of GW2 with CHT14 and PGK but not with GAPDH (Fig. 2a, b). Moreover, in vitro ubiquitination assay revealed that GW2 did not ubiquitinate CHT14 and PGK (Fig. 3a, b). CHT enzymes, which are encoded by a multigene family, degrade chitins and repress pathogen growth (Nakahara and Masuta 2014). This suggests that CHTs play a pivotal role in plant immunity. Direct interaction between GW2 and CHT14 suggests that GW2 can be involved in the protection of the seeds from pathogen attack during development. PGK converts 1,3-bisphosphoglycerate into 3-phosphoglycerate during glycolysis but also participates in the reverse reaction during gluconeogenesis and the Calvin–Benson cycle, indicating that PGK provides the building blocks for carbohydrate biosynthesis (Watson et al. 1982; Brinkmann and Martin 1996). Therefore, direct interaction between GW2 and PGK suggests that GW2 regulates carbohydrate biosynthesis during seed development. Together, these results suggest that the levels of CHT14 and PGK are not directly regulated by the E3 ligase activity of GW2. Nevertheless, direct interactions between GW2 and CHT14 or PGK suggest that GW2 can regulate the activity and level of CHT14 and PGK or, that CHT14 and PGK can control the activity of GW2.

The natural *gw2* mutant Oochikara has a floury endosperm due to small, spherical, and loosely packed starch granules (Song et al. 2007; Choi et al. 2018). This endosperm phenotype of Oochikara is similar to that of the *PDIL1*-*1Δ* mutant; the floury endosperm phenotype of the *pdil1*-*1* deletion mutant is caused by the production of irregular starch granules and protein bodies (Kim et al. 2012). However, in Oochikara, PDIL1-1 accumulated to high levels during the early stages of seed development (Fig. 4a). PDIL ensures proper protein folding through the formation and breakage of intramolecular disulfide bonds (Wilkinson and Gilbert 2004). How a high level of PDIL1-1 protein leads to the production of a floury endosperm is currently unknown. It is possible that PDIL1-1 stabilizes the enzymes involved in branching and debranching of carbohydrates or those involved in formation of protein bodies during early seed development in Oochikara. Failure to degrade these enzymes in a timely manner may lead to their stabilization, thus increasing their activity. Another possibility is that the loss-of-function of *GW2* changes the stability or activity of target proteins involved in endosperm development, including those involved in the formation of starch granules and protein bodies, leading to a floury endosperm. Further in vitro and in vivo analyses are required to elucidate whether PDIL1-1 level and activity are regulated by GW2 through direct interaction between PDIL1-1 and GW2. In addition, the effect of PDIL1-1 on seed phenotypes using transgenic Norin 22 and Oochikara plants overexpressing *PDIL1*-*1* also needs to be investigated.

Investigation of gene expression in *GW2*_*pro*_-*GUS* and *GW2*_*pro*_-*GFP* transgenic rice plants showed that *GUS* was highly expressed in leaves and roots but weakly in leaf sheaths and internodes (Fig. 5a). Notably, *GFP* was highly expressed in the aleurone layer of seeds (Fig. 5b). Additionally, *GW2* expression has been previously demonstrated in various floral organs and seeds (Song et al. 2007; Choi et al. 2018). All of these data strongly indicate that *GW2* regulates rice growth and development during vegetative as well as reproductive growth.

## Conclusions

Overall, we conclude that GW2 strongly interacts with CHT14 and PGK but does not ubiquitinate these proteins. Our data also suggest that CHT14 and PGK can participate in the regulation of the function of GW2, and that GW2 controls seed size through the regulation of CHT14 and PGK levels or activities as well as its E3 ubiquitin ligase activity. Further analysis of transgenic plants will provide insights into how CHT14, PGK, and PDIL1-1 participate in GW2-mediated regulation of vegetative and reproductive growth of rice plants.

## Methods

### Plant material and growth conditions

The natural *japonica* rice *gw2* mutant Oochikara (Accession No. 54075) was obtained from the National Institute of Agrobiological Sciences (NIAS), Japan, and the wild-type rice cultivar Norin 22 was kindly provided by Dr. Hee Jong Koh, Seoul National University. Rice plants were grown at 26 °C under long day conditions (16 h light/8 h dark) in the greenhouse or field.

### 2D PAGE and MALDI-TOF MS

Total proteins were extracted from the seeds of ‘Norin 22’ and ‘Oochikara’ using homogenizing buffer (7 M urea, 2 M thiourea, 4% CHAPS, 1% DTT, 2% pharmalyte, and 1 mM benzamidine). Protein concentrations were determined using the Bradford assay (Bio-Rad, Bradford 1976), and proteins were subjected to 2D PAGE as described previously (Yeu et al. 2007). The signal intensity of each spot was normalized relative to the total spot intensity. Three protein spots, which accumulated to high levels in *gw2* mutant seeds, were subjected to in-gel digestion using modified porcine trypsin. Proteins were identified using an Ettan MALDI-TOF (Amersham Biosciences) as described previously (Yeu et al. 2007).

### Construction of recombinant plasmids

To produce MBP-GW2, GST-CHT14, GST-GAPDH, and GST-PGK fusion proteins, cDNA sequences encoding full-length GW2, CHT14, GAPDH, and PGK were PCR amplified using gene-specific primers and cloned into pMALc2 (New England Biolabs) or pGEX4T-1 (Amersham Biosciences) vectors. Recombinant plasmids were transformed into *E. coli* strain BL21 (DE3) pLysS, and the expression of recombinant proteins was induced with the addition of 5 mM isopropyl-β-d-thiogalactoside (IPTG).

### Purification of recombinant proteins

Recombinant proteins expressed in *E. coli* BL21 cells were purified according to the manufacturer’s instructions. Cells expressing MBP or MBP-GW2 were resuspended and sonicated in a buffer containing 20 mM Tris–HCl (pH 7.4), 200 mM NaCl, 1 mM EDTA, 1% Triton X-100, 2 mM PMSF, and proteinase inhibitor cocktail (Roche). Total protein extracts were loaded onto a column packed with amylose resin (New England Biolabs). Columns were washed with buffer containing 20 mM Tris–HCl (pH 7.4), 200 mM NaCl, 1 mM EDTA, and 1% Triton X-100, and MBP and MBP-GW2 recombinant proteins were eluted with 10 mM maltose.

To purify GST fusion proteins, *E. coli* cells expressing GST-CHT14, GST-GAPDH, and GST-PGK were resuspended and sonicated in buffer containing 50 mM Tris, 50 mM NaCl, 1 mM EDTA, 1% Triton X-100, 2 mM PMSF, and proteinase inhibitor cocktail (Roche). Total protein extracts were loaded onto a column packed with glutathione resin (Pharmacia), and columns were washed with buffer containing 50 mM Tris, 50 mM NaCl, 1 mM EDTA, and 1% Triton X-100. The recombinant proteins GST-CHT14, GST-GAPDH, and GST-PGK were eluted using 10 mM glutathione. All protein concentrations were determined using the Bradford assay (Bio-Rad).

### In vitro pull-down assays

Interactions of MBP-GW2 with GST-CHT14, GST-PGK, and GST-GAPDH were examined using in vitro pull-down assay. Two micrograms of MBP-GW2 was mixed with 2 µg of GST-PGK, GST-CHT14, or GST-GAPDH, and this mixture was added to the reaction buffer containing 50 mM Tris–HCl (pH 7.5), 100 mM NaCl, 1% Triton X-100, 0.2% glycerol, and 0.5 mM 2-mercaptoethanol. Reaction mixtures were incubated at 25 °C for 2 h and then purified using the amylose resin. Resins were washed with buffer containing 50 mM Tris–HCl (pH 7.5), 100 mM NaCl, and 1% Triton X-100 five times. Proteins were eluted using 10 mM glutathione and separated by 10% SDS-PAGE. The GST fusion proteins were detected by immunoblotting using anti-GST antibody (0.4 μg ml^−1^; Santa Cruz Biotechnology).

### In vitro ubiquitination assay

In vitro ubiquitination reactions were carried out in reaction buffer containing 50 mM Tris–HCl (pH 7.4), 5 mM MgCl_2_, 150 mM NaCl, 2 mM ATP, and 1 mM DTT. Each reaction mixture contained 50 ng of E1 (Boston Biochem), 100 ng of E2 (Boston Biochem), 5 µg of His_6_-ubiquitin (Sigma), 200 ng of MBP-GW2, and 100 ng of GST-CHT14 or GST-PGK. Reactions were incubated at 30 °C for 3 h, and proteins were separated by 10% SDS-PAGE. Ubiquitination of GST-CHT14 and GST-PGK was examined by immunoblotting with anti-GST antibody (0.4 μg ml^−1^; Santa Cruz Biotechnology).

### Promoter activity analysis

Transgenic rice lines overexpressing *GW2*_*pro*_-*GUS* and *GW2*_*pro*_-*GFP* were generated as described previously (Choi et al. 2018). Histochemical GUS staining assays were carried out as described previously (Shomura et al. 2008). Specimens were photographed using the OLYMPUS SZX7 microscope. GFP fluorescence in flowers and immature seeds of transgenic and wild-type rice plants was observed using a fluorescence microscope (LAS 4000 imager, GE Life Sciences).

### Western blot analysis

The level of PDIL1-1 protein during seed development was examined in wild-type and *gw2* mutant rice seeds harvested at 5, 10, 20, and 30 DAF. Total proteins were extracted from whole grains using buffer containing 50 mM Tris–Cl (pH 7.5), 150 mM NaCl, 1% Triton X-100, and 1 mM PMSF, and then separated by 10% SDS-PAGE. The PDIL1-1 protein was detected by immunoblotting with anti-PDIL1-1 antibody as described previously (Kim et al. 2012).

## Abbreviations

CHT14: chitinase
*E. coli*: *Escherichia coli*
EXPLA1: EXPANSIN-LIKE 1
GAPDH: glyceraldehyde 3-phosphate dehydrogenase
GFP: green fluorescence protein
GST: glutathione *S*-transferase
GUS: β-glucuronidase
GW2: grain width 2
MALDI-TOF: matrix assisted laser desorption/ionization time-of-flight mass spectrometry
MBP: maltose binding protein
PAGE: polyacrylamide gel electrophoresis
PDIL1-1: protein disulfide isomerase-like 1-1
PGK: phosphoglycerate kinase
RING: real interesting new gene
RT-PCR: real time polymerase chain reaction
SDS: sodium dodecyl sulfate
